# Hepatitis D Virus Entry Inhibitors Based on Repurposing Intestinal Bile Acid Reabsorption Inhibitors

**DOI:** 10.3390/v13040666

**Published:** 2021-04-12

**Authors:** Michael Kirstgen, Kira Alessandra Alicia Theresa Lowjaga, Simon Franz Müller, Nora Goldmann, Felix Lehmann, Dieter Glebe, Karl-Heinz Baringhaus, Joachim Geyer

**Affiliations:** 1 Institute of Pharmacology and Toxicology, Biomedical Research Center Seltersberg (BFS), Faculty of Veterinary Medicine, Justus Liebig University Giessen, Schubertstr. 81, 35392 Giessen, Germany; michael.kirstgen@vetmed.uni-giessen.de (M.K.); Kira.A.Lowjaga@vetmed.uni-giessen.de (K.A.A.T.L.); Simon.Mueller@vetmed.uni-giessen.de (S.F.M.); 2 Institute of Medical Virology, National Reference Center for Hepatitis B Viruses and Hepatitis D Viruses, Justus Liebig University Giessen, 35392 Giessen, Germany; Nora.Goldmann@viro.med.uni-giessen.de (N.G.); Felix.Lehmann@viro.med.uni-giessen.de (F.L.); Dieter.Glebe@viro.med.uni-giessen.de (D.G.); 3 German Center for Infection Research (DZIF), Giessen-Marburg-Langen Partner Site, 35392 Giessen, Germany; 4 Sanofi-Aventis Deutschland GmbH, 65926 Frankfurt, Germany; Karl-Heinz.Baringhaus@sanofi.com

**Keywords:** HBV, HDV, NTCP, ASBT, bile acid transport, entry inhibitor, structure-activity relationship

## Abstract

Identification of Na^+^/taurocholate co-transporting polypeptide (NTCP) as high-affinity hepatic entry receptor for the Hepatitis B and D viruses (HBV/HDV) opened the field for target-based development of cell-entry inhibitors. However, most of the HBV/HDV entry inhibitors identified so far also interfere with the physiological bile acid transporter function of NTCP. The present study aimed to identify more virus-selective inhibitors of NTCP by screening of 87 propanolamine derivatives from the former development of intestinal bile acid reabsorption inhibitors (BARIs), which interact with the NTCP-homologous intestinal apical sodium-dependent bile acid transporter (ASBT). In NTCP-HEK293 cells, the ability of these compounds to block the HBV/HDV-derived preS1-peptide binding to NTCP (virus receptor function) as well as the taurocholic acid transport via NTCP (bile acid transporter function) were analyzed in parallel. Hits were subsequently validated by performing in vitro HDV infection experiments in NTCP-HepG2 cells. The most potent compounds S985852, A000295231, and S973509 showed in vitro anti-HDV activities with IC_50_ values of 15, 40, and 70 µM, respectively, while the taurocholic acid uptake inhibition occurred at much higher IC_50_ values of 24, 780, and 490 µM, respectively. In conclusion, repurposing of compounds from the BARI class as novel HBV/HDV entry inhibitors seems possible and even enables certain virus selectivity based on structure-activity relationships.

## 1. Introduction

Hepatitis B (HBV) and D (HDV) virus infections are the main cause of hepatocellular carcinoma (HCC) and liver cirrhosis as consequences of chronic hepatitis. Available vaccination cannot change that more than 250 million people worldwide currently suffer from HBV/HDV chronic infections, which are associated with more than 800,000 deaths annually [1]. The 3.2 kb comprising genome of the enveloped DNA virus HBV encodes for three envelope proteins, referred to as small (SHBs), middle (MHBs), and large (LHBs) [2]. The myristoylated preS1 domain (myr-preS1_2-48_ lipopeptide), constituting the 2-48 N-terminal amino acids of the LHBs, is essential for virus binding to its hepatic receptor [3,4]. As an HBV satellite virus, HDV is coated with the identical HBV envelope proteins. Therefore, HBV and HDV virus binding to hepatocytes shares the identical mechanism [5]. Approximately 5% of all chronic HBV carriers are superinfected with HDV [1]. This results in more rapid disease progression, increased mortality rates, and increased incidence of HCC and liver cirrhosis [6]. Nucleoside reverse transcriptase inhibitors and interferon are used as a common therapy to keep HBV/HDV associated chronic hepatitis under control. Unfortunately, this therapy rarely leads to curation. Furthermore, interferon therapy is highly prone to adverse drug reactions and nucleoside reverse transcriptase inhibitors have to be given life-long [7,8].

An appropriate drug target to prevent HBV/HDV virus entry into hepatocytes is represented by the Na^+^/taurocholate co-transporting polypeptide NTCP (gene symbol *SLC10A1*). In 2012, NTCP was identified as the bona fide hepatic receptor for HBV/HDV [9,10]. After attachment of the HBV/HDV virus particles to heparan sulfate proteoglycans, the high-affinity binding to NTCP via the virus myr-preS1_2-48_ lipopeptide is essential for virus entry [11,12]. This myr-preS1_2-48_ lipopeptide itself has the ability to block HBV/HDV binding to NTCP and infection with inhibitory constants (IC_50_) in the low nanomolar range [4]. Based on this mechanism, the synthetic myr-preS1_2-48_ lipopeptide analog Hepcludex**^®^** (MYR GmbH, Bad Homburg, Germany) was developed and approved as the first-in-class HDV entry inhibitor [13]. This opened the door for the development of further HBV/HDV entry inhibitors, preferably based on small molecules with oral bioavailability and low impact on the physiological bile acid transporter function of NTCP [14]. In a previous study, we could demonstrate that small molecules from the group of pentacyclic triterpenoids show anti-HDV activity in vitro. An additional desired effect was a certain selectivity for blocking of the virus receptor function of NTCP, while interference with the physiological bile acid transport function only occurred at very high inhibitor concentrations [15].

In search of further appropriate HBV/HDV entry inhibitor drug candidates, the present study focused on inhibitors of the apical sodium-dependent bile acid transporter (ASBT, gene symbol *SLC10A2*). As an additional member of the solute carrier family SLC10, ASBT represents the intestinal counterpart to NTCP for the maintenance of the enterohepatic circulation of bile acids [14]. ASBT is localized at the apical brush border membrane of ileal enterocytes and here is essential for the reabsorption of bile acids from the intestinal lumen [16]. Several inhibitors of ASBT have been developed and approved as bile acid reabsorption inhibitors (BARIs), including elobixibat, odevixibat, linerixibat, volixibat, and maralixibat. These drugs prevent bile acid reabsorption from the gut, reduce bile acid reflux to the liver and increase the hepatic de novo bile acid synthesis from cholesterol. In the end, this has an LDL cholesterol-lowering effect [17]. The present study aimed to repurpose BARIs as novel HBV/HDV entry inhibitors based on the close phylogenetic relationship and functional homology of ASBT and NTCP [18]. Therefore, 87 compounds from the BARI chemical class of propanolamines were screened for blocking of the virus receptor and bile acid transporter functions of NTCP. We could show structure-activity relationships (SAR) for some of the compounds regarding efficient blocking of myr-preS1_2-48_ lipopeptide binding to NTCP. Some compounds even showed certain virus-selectivity and blocked the physiological bile acid transport function only at very high inhibitor concentrations. Based on the results of the present study, repurposing of compounds from the BARI class as novel HBV/HDV entry inhibitors seems possible and even enables certain virus selectivity based on SAR.

## 2. Materials and Methods

### 2.1. NTCP-Expressing Cell Lines

Human embryonic kidney (HEK293) cells were stably transfected with human NTCP, C-terminally tagged with the FLAG epitope (further referred to as NTCP-HEK293 cells) as reported before [19]. Cells were maintained at 37 °C, 5% CO_2_, and 95% humidity in DMEM/F-12 medium (Thermo Fisher Scientific, Waltham, MA, USA) supplemented with 10% fetal calf serum (Sigma-Aldrich, St. Louis, MO, USA), 4 mM L-glutamine (PAA, Cölbe, Germany) and penicillin/streptomycin (PAA). HepG2 cells stably transfected with NTCP-FLAG (further referred to as NTCP-HepG2 [10]) were cultured under the same conditions in DMEM with all supplements listed above, except for L-glutamine. For induction of the transgene, the medium was supplemented with 1 µg/mL tetracycline (Roth, Karlsruhe, Germany) in the case of the NTCP-HEK293 cells or with 2 µg/mL doxycycline (Sigma-Aldrich) in the case of the NTCP-HepG2 cells.

### 2.2. Inhibitory Concentrations (IC_50_) for [^3^H]preS1 Binding and [^3^H]Taurocholic Acid Transport

Bile acid transport measurements were performed in NTCP-HEK293 cells as described [15]. Briefly, [^3^H]taurocholic acid, further referred to as [^3^H]TC, (20 Ci/mmol, 0.09 mCi/mL, Perkin Elmer, Waltham, MA, USA) was used as substrate. In parallel, [^3^H]preS1 peptide binding experiments were performed with a tritium-labelled myr-preS1_2-48_ lipopeptide -HBV subgenotype D3- that was purchased from Pharmaron (120 Ci/mmol, 1 mCi/mL, Cardiff, UK). Cells were seeded onto polylysine-coated 96-well plates, induced with 1 µg tetracycline per ml, and grown to confluence over 72 h at 37 °C. Then, cells were washed once with tempered phosphate buffered saline (PBS, 137 mM NaCl, 2.7 mM KCl, 1.5 mM KH_2_PO_4_, 7.3 mM Na_2_HPO_4_, pH 7.4) at 37 °C, and pre-incubated with 80 µL DMEM for 5 min at 37 °C. The medium was replaced by 80 µL DMEM containing the respective inhibitor (concentrations ranging from 0.1 to 1000 µM) or solvent alone (100% uptake/binding control), and cells were further incubated for 5 min at 37 °C. After pre-incubation, bile acid transport experiments were started by adding 20 µL DMEM containing 5 µM [^3^H] TC (final concentration: 1 µM). Binding of [^3^H]preS1 was initiated by adding 20 µL DMEM containing 25 nM [^3^H]preS1 (final concentration: 5 nM). Experiments were stopped after 10 min by two-times washing with ice-cold PBS. For 0% uptake/binding control, the NTCP-HEK293 cells were not induced with tetracycline (-tet). Cell-associated radioactivity of [^3^H]TC or [^3^H]preS1 was quantified by liquid scintillation counting in a Packard Microplate Scintillation Counter TopCount NXT (Packard Instrument Company, Meriden, CT, USA). Transport rates and [^3^H]preS1 binding were determined in counts per minute (cpm). The mean of the 0% control was subtracted and the net [^3^H]TC transport rates and net [^3^H]preS1 binding rates, respectively, were expressed as percentage of control. A set of 87 test compounds was provided by Sanofi-Aventis Deutschland GmbH (Frankfurt, Germany). IC_50_ values were calculated from quadruplicate determinations by GraphPad Prism 6 (GraphPad, San Diego, CA, USA).

### 2.3. HDV Infection Experiments

HDV production was done in vitro as described before [20,21]. RT-qPCR was performed to determine genome equivalents. NTCP-HepG2 cells were pre-incubated for 5 min with inhibitors solved in 80 µL HGM per well in concentrations ranging from 5 µM to 300 µM. Infection experiments were performed in NTCP-HepG2 cells as described [15]. Briefly, during infection, cells were cultured in 96-well plates in Hepatocyte Growth Medium (HGM) consisting of William’s E Medium (Thermo Fisher Scientific) containing 2% bovine serum albumin (BSA, Roth), 2 mM L-glutamine (Thermo Fisher Scientific), 100 µg/mL gentamicin (Thermo Fisher Scientific), 10 nM dexamethasone (Sigma-Aldrich), 1 mM sodium pyruvate (Thermo Fisher Scientific), 1 × Insulin-Transferrin-Selen (Thermo Fisher Scientific), 2% DMSO (Merck, Darmstadt, Germany), 4% polyethylene glycol (Sigma-Aldrich), and 2 µg/mL doxycycline (Sigma-Aldrich). HDV stock solved in 20 µL HGM per well was added for infection and cells were incubated for 6 h with a final concentration of 120 genome equivalents/cell of HDV particles. Subsequently, cells were washed with DMEM and cultured in HGM supplemented with 2% DMSO, 2% BSA and 2 µg/mL doxycycline. Every three days, medium was changed until cells were fixed at 9 days post infection with 3% paraformaldehyde (Sigma-Aldrich) in PBS, for 30 min at room temperature (RT). Cells were permeabilized with 0.2% Triton X 100 (Roth) in PBS for 30 min at RT, and blocked by incubation with 5% bovine serum albumin (Roth) in PBS for 30 min at RT. Then, cells were immunostained with purified human anti-HDV-positive serum at 37 °C for 1 h (1:400 dilution). Goat anti-human IgG secondary antibody coupled to Alexa Fluor fluorophore (1:400 dilution, Thermo Fisher Scientific) was added for 1 h at 37 °C for detection of Hepatitis Delta antigen (HDAg) as described before [22]. Nuclei were stained with Hoechst 33342 (1 µg/mL, Thermo Fisher Scientific).

### 2.4. Cytotoxicity Assay

In Vitro Toxicology Assay Kit (Sigma-Aldrich) was used to perform a 3-[4,5-dimethylthiazole-2-yl]-2,5-diphenyltetrazolium bromide (MTT) assay to measure the cytotoxicity of the indicated compounds according to the manufacturer’s protocol. Briefly, NTCP-HepG2 cells were incubated with 100 µL of the indicated concentrations of the respective compound solved in HGM over 6 h at 37 °C. After 6 h, medium was replaced by inhibitor-free HGM and cells were cultured for additional 24 h. Then, medium was removed and 100 µL DMEM containing 0.5 mg/mL MTT were added and cells were incubated for 1 h at 37 °C. Finally, the medium was replaced by 100 µL isopropyl alcohol (Sigma-Aldrich) and samples were measured by ELISA reader (GloMax-Multi Detection System, Promega, Madison, WI, USA).

### 2.5. Structure Modeling

The 2D compound structures were generated from an SDfile, associated to the tested compounds and provided by Sanofi-Aventis Deutschland GmbH, using the MAESTRO 12.2 Molecular Modeling Interface of SCHRÖDINGER, Inc. (New York City, NY, USA).

### 2.6. Statistics

Determination of IC_50_ values was done by nonlinear regression analysis using the equation log(inhibitor) vs. response settings of the GraphPad Prism 6.0 software (GraphPad). Data of bile acid transport and myr-preS1_2-48_ lipopeptide binding are expressed as means ± SD from quadruplicate determinations. IC_50_ values are listed with their 95% confidence intervals in Table 1. In addition, mean IC_50_ values were calculated for each experiment. Cytotoxicity studies represent data from two independent experiments, each with quadruplicate determinations. Statistical analysis was done by one-way ANOVA, followed by Dunnett’s multiple comparison test by GraphPad Prism 6.0, considering *p* < 0.01 as statistically significant. Infection studies show data from three independent experiments, each with triplicate determinations represented as means ± SEM. Statistical analyses of the HDV infection experiments were performed by two-way ANOVA, followed by Dunnett’s multiple comparison test by GraphPad Prism 6.0, considering *p* < 0.01 as statistically significant.

## 3. Results

**Figure 1:**
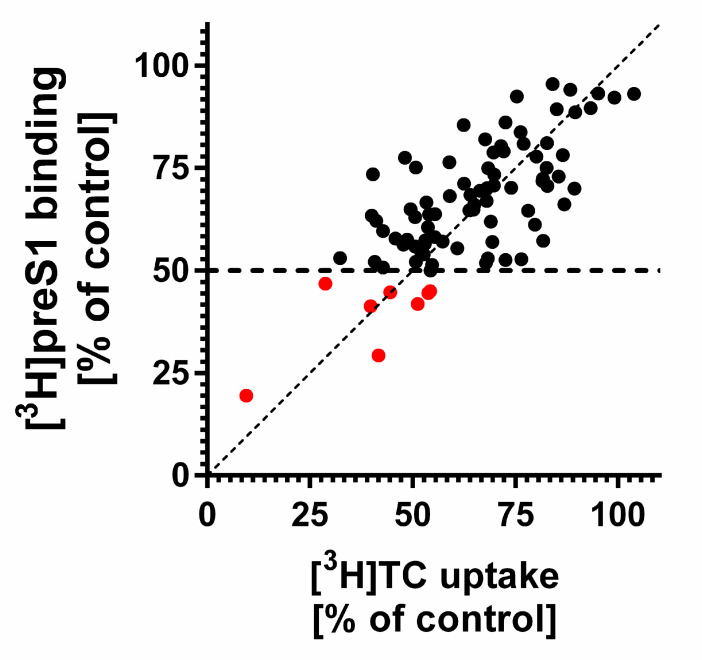
Inhibitory potency of 87 propanolamine derivatives on [^3^H]TC uptake via NTCP and [^3^H]preS1 lipopeptide binding to NTCP. NTCP-HEK293 cells were seeded onto 96-well plates and were incubated with tetracycline to induce expression of NTCP. Experiments were performed in the presence of 100 µM inhibitor to block uptake of 1 µM [^3^H]TC (shown at the *x*-axis) and binding of 5 nM [^3^H]preS1 (shown at the *y*-axis) over 10 min at 37 °C. Control experiments were performed with solvent alone (set to 100%). Red dots represent the compounds S985852, A000028897, A000295480, S973515, A000289041, S973509, A000295231, and A000295013, which showed <50% remaining [^3^H]preS1 binding compared to untreated control. Bisector is illustrated as dashed line. Compounds below the bisector line represent superior inhibitors of [^3^H]preS1 lipopeptide binding compared to [^3^H]TC uptake inhibition.

**Figure 2:**
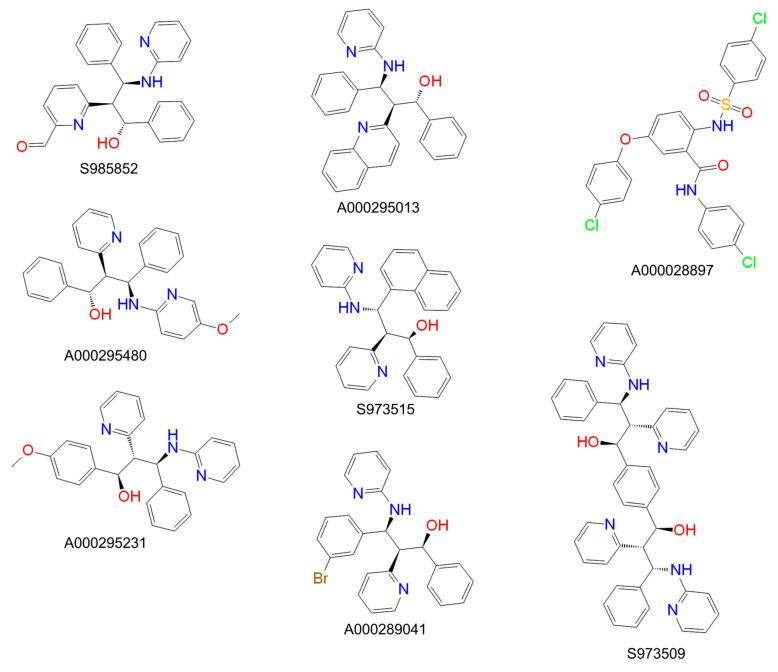
Structures of the eight most potent propanolamine [^3^H]preS1 lipopeptide binding inhibitors (see Figure 1).

For inhibition of myr-preS1_2-48_ lipopeptide binding to NTCP, 87 propanolamine derivatives were used as potential inhibitors. These derived from a previous development of ASBT-inhibiting BARIs at Sanofi-Aventis Deutschland GmbH. In a first step, all compounds were used at 100 µM inhibitory concentration in transport / binding assays with 1 µM [^3^H]TC / 5 nM [^3^H]preS1 (Figure 1). The best performing compounds from this pre-screen were defined as those with <50% remaining [^3^H]preS1 binding in the presence of inhibitor compared to untreated control. In total, eight compounds fulfilled this condition (red dots in Figure 1). All of them are structurally presented in Figure 2.

**Figure 3:**
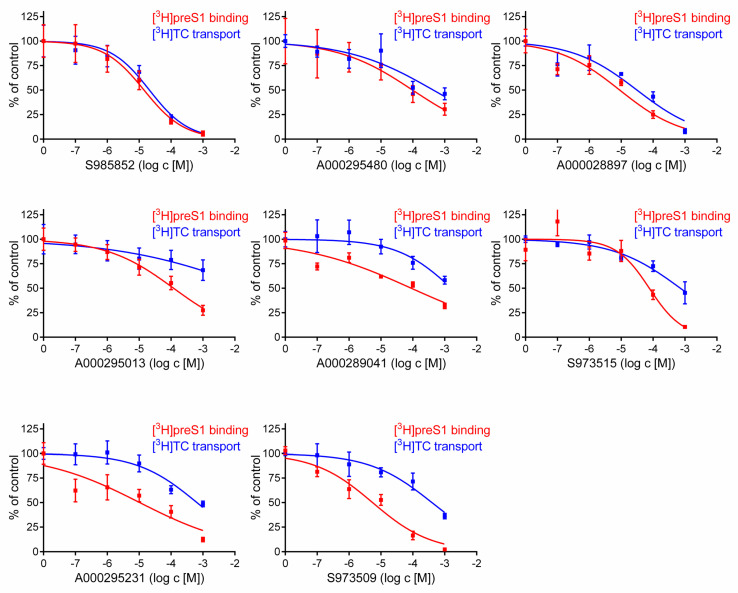
Inhibition of [^3^H]preS1 peptide binding to NTCP and [^3^H]TC transport via NTCP at increasing concentrations of propanolamine derivatives. NTCP-HEK293 cells were seeded onto 96-well plates and were incubated with tetracycline to induce expression of NTCP. Cells without tetracycline treatment were used as 0% controls for both assays. Bile acid transport experiments were performed with 1 µM [^3^H]TC and binding experiments were performed with 5 nM [^3^H]preS1. Both assays were performed over 10 min at 37 °C with increasing concentrations of the indicated inhibitors. Control experiments were performed with solvent alone (set to 100%). The mean of the 0% control was subtracted to calculate net [^3^H]TC transport rates (shown in blue) as well as net [^3^H]preS1 binding rates (shown in red), which are expressed as % of control at the *y*-axis. Half-maximal inhibitory concentrations (IC_50_) were calculated by nonlinear regression analysis using the equation log(inhibitor) vs. response (GraphPad Prism). Data represent means ± SD of quadruplicate determinations of representative experiments.

For these eight compounds, detailed inhibitory assays were performed with inhibitor concentrations ranging from 0.1 µM to 1000 µM, in order to determine IC_50_ values for inhibition of 1 µM [^3^H]TC transport via NTCP and 5 nM [^3^H]preS1 binding to NTCP (Figure 3). Resulting IC_50_ values are listed as 95% confidence intervals in Table 1. As an additional variable for interpretation of the inhibitory pattern of the compounds, a selectivity index was implemented. This index is the non-dimensional quotient from the IC_50_ values for [^3^H]TC uptake and [^3^H]preS1 binding inhibition and describes the degree of virus/preS1 selectivity. In this case, the myr-preS1_2-48_ lipopeptide binding represents a surrogate for HBV/HDV binding to NTCP. The higher the selectivity index, the higher the inhibitory impact on myr-preS1_2-48_ lipopeptide binding of the tested compound compared to the inhibitory impact on taurocholic acid uptake.

The IC_50_ values for [^3^H]TC transport inhibition ranged from 21 up to >1000 µM and the IC_50_ values for [^3^H]preS1 peptide inhibition ranged from 14 up to 119 µM, but without any significant correlation, indicating that both functions of NTCP are differently addressed by these propanolamine inhibitors. Interestingly, the compounds significantly differed in their virus/preS1 selectivity. While three compounds (S985852, A000295480, and A000028897) were more or less non-selective, others were more selective for preS1-peptide inhibition, being S973509, A000295231, and A000295013, with selectivity indices of 53, 65, 313, respectively (Figure 3, Table 1). However, the selectivity index of 313 for compound A000295013 must not be overestimated. This high index is the result of an undeterminable IC_50_ value for [^3^H]TC uptake inhibition (IC_50_ > 1000 µM) in combination with a quite low activity against [^3^H]preS1-peptide binding with IC_50_ of approximately 130 µM. Consequently, this compound represents a potentially selective but not potent inhibitor of myr-preS1_2-48_ lipopeptide binding. Furthermore, the observed IC_50_ values of the compounds A000295480, S973515, and A000289041 for [^3^H]preS1 binding inhibition were relatively high, what made these compounds less attractive for further investigation.

**Figure 4:**
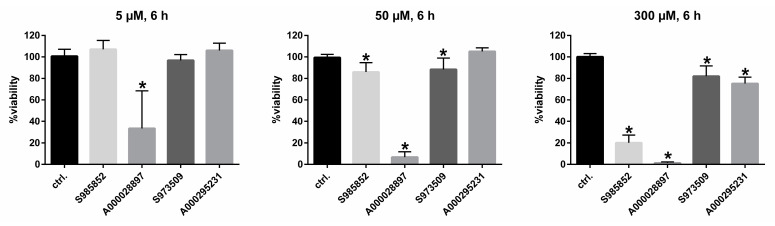
Cytotoxicity studies in NTCP-HepG2 cells. An MTT cytotoxicity assay was performed with the indicated propanolamine derivatives at 5 µM, 50 µM, and 300 µM inhibitor concentrations, incubated over a time span of 6 h at 37 °C. Solvent control was set to 100%. Data represent means ± SD of two independent experiments, each with quadruplicate determinations (*n* = 8). * Significantly different to control with *p* < 0.01.

Based on this, for subsequent in vitro infection studies, we selected compounds S985852 and A000028897 as potent but non-selective inhibitors as well as compounds S973509 and A000295231 as potent and selective inhibitors of viral preS1-binding to NTCP. Next, cytotoxicity of these compounds on NTCP-overexpressing HepG2 cells, being the model cell line for in vitro HDV infection experiments, was analyzed at low (5 µM), middle (50 µM), and high (300 µM) inhibitor concentrations. Corresponding to the time-schedule for in vitro HDV infection, these cytotoxicity assays were performed for a 6 h time span of compound incubation (Figure 4).

As indicated in Figure 4, compound A000028897 was excluded at this point due to obvious cytotoxicity even at low (5 µM) concentrations. All other compounds did not show strong cytotoxic effects at concentrations of 5 and 50 µM. However, compound S985852 showed critical cytotoxicity at 300 µM, which is way above the determined IC_50_ value for [^3^H]preS1 binding inhibition (being 8–24 µM), so this compound was not excluded for infection experiments.

**Figure 5:**
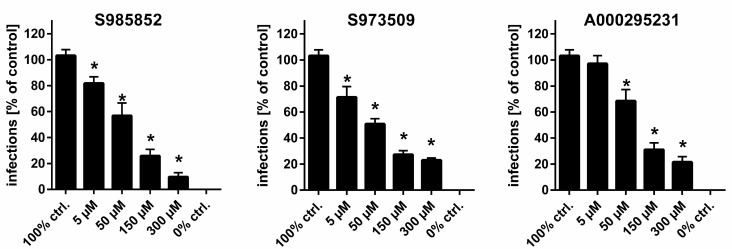
HDV infection studies. NTCP-HepG2 cells were pre-incubated for 5 min with the indicated concentrations of the indicated inhibitors in DMEM at 37 °C. Then, cells were additionally inoculated with 120 genome equivalents/cell of HDV particles at 37 °C. After 6 h, cells were washed and further incubated with inhibitor- and virus-free medium, and medium was changed every 3–4 days. At day 9 post infection, cells were fixed and an immunostaining against the HDAg was performed, as a marker of HDV infection. The number of infected cells per well was determined by fluorescence microscopy. NTCP-HepG2 cells incubated without inhibitor were used as control (set to 100% infection rate). Infection experiments in the presence of 0.5 µM myr-preS1_2-48_ lipopeptide served as 0% control (representing 0% infection rate). Data represent means ± SEM of three independent experiments, each with triplicate determinations (*n* = 9). * Significantly different to 100% control wit *p* < 0.01.

The remaining three compounds were tested for their potency to block in vitro HDV infection of NTCP-overexpressing HepG2 cells. Compounds S985852, S973509, and A000295231 showed a significant concentration-dependent inhibition of in vitro HDV infection (Figure 5), thereby confirming the pre-screen with the myr-preS1_2-48_ lipopeptide (Figure 3). Of note, the IC_50_ values for [^3^H]preS1 peptide inhibition and in vitro HDV infection inhibition were within the same range. In particular, compound S985852 yielded identical IC_50_ values of approximately 15 µM for both, the [^3^H]preS1 binding assay and the HDV infection experiments. The respective IC_50_ values of compound S973509 differed by a factor of ten. The pre-screen delivered an IC_50_ of 7 µM, whereas infection studies yielded an IC_50_ of 70 µM. For compound A000295231, IC_50_ values were 17 µM for the [^3^H]preS1 pre-screen and 40 µM for the infection studies.

**Figure 6:**
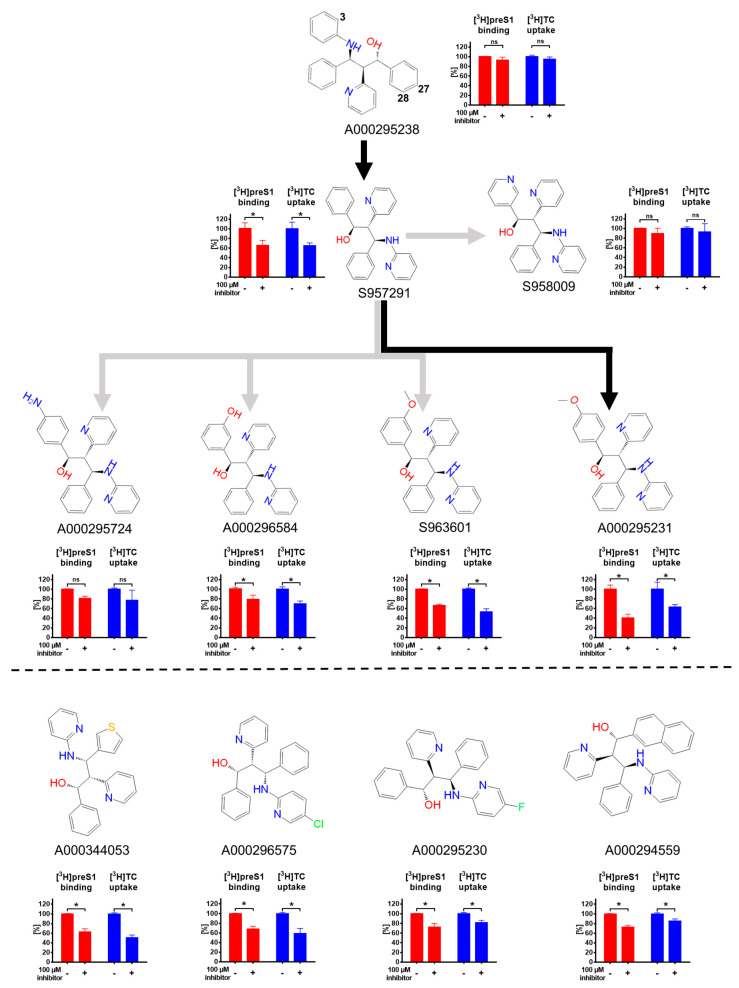
Structure-activity relationships of propanolamine derivatives for [^3^H]TC transport inhibition and [^3^H]preS1 binding inhibition. The respective inhibition pattern are illustrated by bar graphs for [^3^H]TC uptake (shown in blue) and [^3^H]preS1 peptide binding (shown in red) in the presence (+) or absence (−) of a 100 µM inhibitor concentration of the respective propanolamine derivative. Graphs show means ± SD of triplicate determinations. * Significantly different with *p* < 0.01 (two-way ANOVA with Sidak’s multiple comparison test); ns, not significant. Chemical modifications and isomerization of the propanolamine derivatives revealed clear structure-activity relationships for more potent [^3^H]preS1 lipopeptide binding inhibition (A000295231>S957291>A000295238). Several propanolamine derivatives showed significant but less potent [^3^H]preS1 lipopeptide binding inhibition. Four representative compounds (A000344053, A000296575, A000295230, A000294559) are shown.

Finally, we closely analyzed structure-activity relationships (SAR) of selected propanolamine derivatives that can be used for further activity and selectivity optimization (Figure 6). Starting from the inactive propanolamine compound A000295238, isomerization and substitution of C_3_ by nitrogen (compound S957291) significantly increased the inhibitory potency for [^3^H]TC uptake and [^3^H]preS1 binding inhibition. Additional OCH_3_ coupling at C_27_ (compound A000295231) even further improved the inhibitory potency. This compound showed an IC_50_ of ~40 µM for in vitro HDV infection inhibition and selectivity index of 65. Therefore, A000295231 represents the most promising compound of the present study. In contrast, addition of an amino group at the same C_27_ position (compound A000295724) made this compound inactive. The same was true for N-substitution of compound S957291 at position C_28_, resulting in compound S958009. However, coupling of an OH-group or OCH_3_-group at C_28_ (compounds A000296584 and S963601, respectively) resulted in significant, but less active inhibitors of [^3^H]preS1 lipopeptide binding. These compounds were not analyzed more in detail in the present study.

Figure 6 shows the structures of four additional propanolamine inhibitors of the [^3^H]preS1 lipopeptide binding to NTCP. These compounds are characterized by substitutions of a benzyl group by thiophenyl (compound A000344053) or naphthyl groups (compound A000294559) and by Cl (compound A000296575) or F addition (compound A000295230). However, as none of these compounds showed >50% inhibition of [^3^H]preS1 peptide binding, they were not analyzed more in detail in the present study. Taken together, these SAR data clearly indicate that propanolamine derivatives can be modified towards more selective bile acid transport inhibition and towards more potent myr-preS1_2-48_ lipopeptide binding inhibition.

## 4. Discussion

The numbers from the global hepatitis report and the fact that to date only one single approved HDV entry inhibitor (bulevirtide, Hepcludex^®^) is available underline the need of more therapeutics against this serious disease [1,4]. Many recent studies focused on NTCP inhibition in order to prevent virus entry into hepatocytes, using cyclosporine A and other cyclosporine derivatives [23,24,25,26], ezetimibe [10], irbesartan [27], ritonavir [28], (-)-epigallocatechin-3-gallate [29], vanitaracin A [30], Ro41-5253 [31], proanthocyanidin [32], zafirlukast [33], sulfasalazine [33], and Chicago Sky Blue 6B (an azo dye) [33]. Most of these inhibitors represent non-selective inhibitors of NTCP that are prone to increase serum bile acids and to interfere with the bile acid homeostasis [34]. However, there are some hints that both functions of NTCP, the virus receptor function and the bile acid transporter function, can separately be addressed with inhibitors that are more selective [15,25].

In a previous study, we demonstrated that amino acid G158 of the human NTCP is essential for myr-preS1_2-48_ lipopeptide binding to NTCP and in vitro HBV and HDV infection. This amino acid is located at a domain (amino acids 157–165) that was shown before to be important for myr-preS1_2-48_ lipopeptide binding to human NTCP [9,35]. Consequently, G158R NTCP mutants were insusceptible for in vitro HBV/HDV infection, but still transported bile acids. It was hypothesized that the larger amino acid side chain of arginine compared to glycine might sterically preclude myr-preS1_2-48_ lipopeptide binding, while bile acids can still bind to their binding pocket [22].

In addition, we recently identified small molecules from the group of pentacyclic triterpenoids to be potent HDV entry inhibitors with less effect on the bile acid transport function of NTCP [15]. Likewise, Shimura et al. [25] showed for the cyclosporine derivative SCY995 higher potency against in vitro HBV infection than for bile acid transport inhibition. These findings illustrate the possibility to develop entry inhibitors with oral bioavailability that do not strongly interfere with the physiological bile acid transport function of NTCP.

A common approach for identification of novel active compounds against a given therapeutic target is high-throughput screening (HTS). This technique can rapidly generate data of large subsets of molecules using automated experimental assays [36]. However, hit rates of HTS are quite low (0.01%–0.1%), generating immense drug discovery costs [37,38]. The approach of the presented study was therefore more targeted. We considered that inhibitors of other bile acid transporters, especially the intestinal bile acid transporter ASBT, might also be good inhibitors of NTCP and, thereby potential HDV entry inhibitors. In fact, we identified 3 out of 87 propanolamine derivatives (S985852, S973509, A000295231) to be active against in vitro HDV infection. These propanolamine compounds were previously used to optimize the activity of this compound class against ASBT. Of note, two of the compounds (S973509, A000295231) showed even some selectivity towards the virus receptor function of NTCP and interfered with the physiological bile acid transport function of NTCP only at very high inhibitor concentrations. Overall, this represents a hit rate of approximately 3% at this small sample size, which can be described as excellent enrichment compared to classical HTS.

In addition, it can be emphasized that our screening approach, using [^3^H]preS1 binding inhibition as a surrogate for HDV binding inhibition, represents an advantageous tool for identification of new entry inhibitors. Especially, compound S985852 showed identical IC_50_ values for both assays. This clearly demonstrates the predictivity of the [^3^H]preS1 lipopeptide binding assay, but nevertheless, data from the [^3^H]preS1 binding experiments have to be verified by direct infection experiments. Although HBV and HDV share the identical surface proteins and the same mechanism of interaction with NTCP via the myr-preS1_2-48_ lipopeptide [5], future studies should confirm that also HBV entry is inhibited upon treatment with the inhibitors presented in this study. In addition, it would be interesting to study the efficacy of virus entry inhibition in an HBV/HDV coinfection setting.

The use of compounds known to interact with ASBT could provide further advantages for the development of NTCP inhibitors. If an NTCP inhibitor would be transported from the intestinal lumen via ASBT as a substrate, this would increase intestinal absorption and would result in high inhibitor concentrations in the portal blood. Consequently, inhibitor concentrations at the site of NTCP expression at the basolateral membrane of hepatocytes would be relatively high. In theory, when an inhibitor would be a substrate of both carriers, NTCP and ASBT, this would even enable enterohepatic circulation similar to the physiological cycling of bile acids between liver and gut. Based on this, it would be worth analyzing if the identified NTCP inhibitors from the propanolamine class are also substrates of ASBT and/or NTCP. However, it must be considered that inhibition of ASBT also interferes with the reabsorption of bile acids from the intestinal lumen. This could lead to typical side effects of BARIs, being diarrhea, steatorrhea, and abdominal pain [17].

The structure-activity relationships demonstrated in the present study for propanolamine derivatives make clear that even slight chemical modifications of these molecules were decisive for their inhibitory potential and selectivity to interact with the virus receptor function of NTCP. Interestingly, only few structural differences occur between active and inactive compounds as well as between selective and non-selective inhibitors (see Figure 6). Even more interesting, the study demonstrates that molecules, originally designed as ASBT inhibitors, show cross-reactivity with the closely related NTCP carrier and this cross-reactivity can be used in a positive way to discover potential drug candidates for HBV/HDV entry inhibition. Further studies should now investigate the mode of action of selected candidates (competitive vs. non-competitive inhibition; reversible vs. irreversible inhibition; substrate vs. non-substrate inhibitor) and should be extended to all relevant HDV and HBV genotypes. In addition, the effect of these compounds on other liver-associated transport proteins, such as members of the organic anion transporting polypeptide (OATP) family should be considered. Upon successful completion of these studies, an appropriate in vivo model could be used to investigate the in vivo efficacy of the propanolamine NTCP inhibitors.

In conclusion, repurposing of compounds from the BARIs class as novel HDV entry inhibitors seems possible and even enables certain virus selectivity based on structure-activity relationships.

## Figures and Tables

**Table 1:** The indicated compounds were analyzed for [^3^H]TC transport inhibition and [^3^H]preS1 binding inhibition in NTCP-HEK293 cells. IC_50_ values were calculated from a range of five inhibitor concentrations (0.1–1000 µM) and they are listed with their 95% confidence intervals. Selectivity indices for [^3^H]TC transport inhibition/[^3^H]preS1 binding inhibition were calculated from the IC_50_ means. The higher this index, the more selective the respective inhibitor. Selected compounds were additionally tested for in vitro HDV infection in NTCP-HepG2 cells.

Compound	IC_50_ ([^3^H]TC Uptake) [µM] ^a^	IC_50_ ([^3^H]preS1 Binding) [µM] ^b^	Selectivity Index ^c^	In Vitro HDV Infection ^d^
S985852	13 to 34	8 to 24	2	IC_50_ ~ 15 µM
A000028897	16 to 62	3 to 24	3	toxic
A000295480	145 to 1323	32 to 297	4	ND
S973515	413 to 1238	39 to 148	9	ND
A000289041	>1000	35 to 224	18	ND
S973509	202 to 775	3 to 10	53	IC_50_ ~ 70 µM
A000295231	341 to 1221	4 to 29	65	IC_50_ ~ 40 µM
A000295013	>1000	73 to 194	313	ND

^a^ Inhibition of 1 µM [^3^H]TC uptake; ^b^ inhibition of 5 nM [^3^H]preS1 binding; ^c^ calculated from mean IC_50_ TC:preS1; ^d^ see also Figure 4 and Figure 5; ND, not determined.

## Data Availability

Obtained and analyzed data of this study are available from the corresponding author on request. Compounds are accessible on the basis of a material transfer agreement (MTA) and upon availability in the compound collection.
